# Effects of a standard high-fat diet with or without multiple deficiencies on bone parameters in ovariectomized mature rat

**DOI:** 10.1371/journal.pone.0184983

**Published:** 2017-09-26

**Authors:** Ting Wang, Xiaohuan Zhu, Fang Dai, Chaofei Li, Dake Huang, Zhaohui Fang, Qiu Zhang, Yunxia Lu

**Affiliations:** 1 Department of Endocrinology, The First Affiliated Hospital of Anhui Medical University, Hefei, Anhui, People’s Republic of China; 2 Department of Biochemistry and Molecular Biology, College of Basic Medicine, Anhui Medical University, Hefei, Anhui, People’s Republic of China; 3 The Comprehensive Laboratory, College of Basic Medicine, Anhui Medical University, Hefei, Anhui, People’s Republic of China; 4 Department of Endocrinology, The First Affilliated Hospital of Anhui University of Traditional Chinese Medicine, Hefei, Anhui, People’s Republic of China; Nanjing Medical University, CHINA

## Abstract

The aim of this study was to determine the effects of a standard high fat diet (D12451) with or without vitamin D3, phosphorus, and calcium (i.e., high-fat diet [HFD] or high-fat deficient diet [HFDD]) on the bone parameters of ovariectomized female rats. Six-month-old of female Sprauge Dawley (SD) rats were randomly divided into six study groups: sham operation with standard chow diet (SSCD), sham operation with a HFD (SHFD), sham operation with a HFDD (SHFDD), ovariectomized (OVX), OVX with a HFD (OVX-HFD), and OVX with a HFDD (OVX-HFDD). A bilateral ovariectomy was administered to the OVX, OVX-HFD, and OVX-HFDD rats, while the SSCD, SHFD, and SHFDD rats were only given a laparotomy. Multiple analyses concerning the glucose and insulin tolerance, structure, bone strength, bone matrix, and mineralization of the rats were conducted in order to produce a detailed characterization of the effects of a HFD and a HFDD on postmenopausal osteoporotic rats. Seven months of HFD and HFDD feeding resulted in obesity and insulin resistance in female SD rats. A standard HFD increased the bone calcium content and bone strength of OVX rats. Conversely, the serum N-mid osteocalcin (N-MID-OT) and tartrate-resistant acid phosphatase (TRAP) levels in the OVX-HFDD group were increased, accompanied by a clear decrease in the bone mineral density (BMD), bone mineral content (BMC), bone calcium and bone strength, as well as reduced osteocalcin expression. A HFDD weakened the activity of the osteoblasts while aggravating bone loss and decreasing bone strength in ovariectomized rats, which may be due to the calcium, phosphorus and vitamin D3 deficiencies in the diet.

## Introduction

Osteoporosis is a skeletal disease characterized by low bone mass and the structural deterioration of bone, resulting in an increased susceptibility to fractures [1]. An imbalance between bone formation and bone resorption is the predominant mechanism behind osteoporosis [2]. The prevalence of osteoporosis continues to increase, and it will likely become an even more serious public health problem in an aging society [3]. Generally, ovariectomy (OVX) of female rats or mice has been used to mimic human osteoporosis, with the ovariectomized rats/mice eventually developing increased bodyweight, fat mass, and metabolic dysfunction [4,5].

Obesity is another serious public health problem, which is characterized by the excessive accumulation of adipose tissue in the body. In fact, osteoporosis and obesity are complex disorders with a pathophysiological linkage, since both fat and bone cells originate from the same bone marrow stem cells [6, 7]. The relationship between obesity and osteoporosis has therefore been widely studied.

The traditional view is that obesity is detrimental to bone health, and a negative correlation between body weight or body mass index and bone mass has been reported in mice and rats [8, 9]. However, challenging this widely accepted view, numerous studies have provided evidence, indicating that adipose tissue actually serves to protect the skeleton [10, 11]. Furthermore, the protective or detrimental effect is said to be related to the site and type of adipose tissue, which means that subcutaneous adipose tissue has the protective effect and visceral adipose tissue has the detrimental effect on bone structure and strength [12]. High-fat diet (HFD)-induced obesity in animal models is believed to best mimic the physiological functions of an obese human body, although it must be noted that the conflicting results seen in previous studies may be attributable to the different ingredients of the diets, especially the species of fatty acids [13, 14, 15]. Given these discrepancies in the previously published results, further investigation aimed at elucidating the relationship between osteoporosis and obesity is warranted.

Vitamin D deficiency leads to reduced calcium absorption, which is exacerbated by calcium deficiency, thereby disturbing calcium homeostasis as well as increasing bone resorption and bone remodeling rates. A large European study of 8532 postmenopausal women with inadequate vitamin D levels found 97% of participants to be osteoporotic [16]. Another study reported that 82% of osteoporotic patients exhibited inadequate calcium intake, consuming less than the recommended dosage of 1000 mg per day [17]. Furthermore, phosphorus deficiency is yet another marker of generally nutritional inadequacy in elderly patients, which causes an increased fracture risk [18]. Hence, deficiencies in vitamin D, phosphorus, and calcium aggravate the chance of fractures in elderly osteoporotic women, although there have been no reports concerning the influence of a high-fat diet with a common lack of those three components on osteoporosis.

The standard HFD (D12451, 45% energy from fat; Research Diets, Inc., New Brunswick, NJ, USA) is a commonly accepted high-fat diet used for the development of obesity in mice and rats [19]. It is also considered to be “comprehensive” because of its general ingredients, including carbohydrate, lipid and protein, as well as a multiple vitamin and mineral mixture. We hypothesized that osteoporotic rats fed with D12451 will exhibit an increase in their body mass and an improvement in the microstructural and mechanical properties of their long bones, although these protective effects will vanish when vitamin D, phosphorus and calcium are deleted from the D12451. Therefore, in this study, we chose a D12451 with or without vitamin D3, phosphorus, and calcium (HFD or high-fat deficient diet [HFDD]) to feed sham and bilateral ovariectomized rats in order to observe the beneficial effects of a HFD and the deleterious effects of a HFDD on the bone parameters of female rats with postmenopausal osteoporosis (PMOP). To the best of our knowledge, this is the first study to present a comparison of the effects of a comprehensive HFD with those of HFDD on a 13-month-old female osteoporotic rat model.

## Materials and methods

### Animals and treatment

Thirty-six 6-month-old female Sprague-Dawley (SD) rats were purchased from the Experimental Animal Center at Anhui Medical University. The rats were housed in plastic cages (six rats/cage) and had free access to water and food, and they were kept in conditions of controlled temperature (22±1°C) and lighting (12-h light/dark cycle) for one week in order to acclimatize. The rats were randomized into six study groups (n = six/group), sham operation with a standard chow diet (SSCD), sham-operated with a HFD (SHFD), sham operation with a HFDD (SHFDD), ovariectomized (OVX), OVX with a HFD (OVX-HFD), and OVX with a HFDD (OVX-HFDD). The rats in the SSCD, SHFD, and SHFDD groups underwent a laparotomy, while the rats in the OVX, OVX-HFD, and OVX-HFDD groups were given a bilateral ovariectomy. The SSCD and OVX rats received normal feeding, whereas the SHFD, SHFDD, OVX-HFD, and OVX-HFDD rats were fed with either a HFD or a HFDD for seven months after surgery. There are no caloric differences between the HFD and the HFDD. The normal feed (D12450B), HFD and HFDD were all self-made according to recipes published by Research Diets. The nutritional composition and the composition of the vitamin and mineral mixes in the D12450B and D12451 were described in [19]. All the rats underwent the subcutaneous injection of tetracycline (25 mg/kg) on days 14 and 13, and calcein (10 mg/kg, both from Sigma) on days four and three prior to sacrifice. Then, the glucose tolerance test and insulin tolerance test were performed to detect insulin sensitivity one week before sacrifice. All the experiments were approved by the Ethics Committee of Anhui Medical University.

### Sample collection and applications

The rats were weighed every week. Food intake was measured by weighing the food consumption in every cage each day divided by the numbers of rats in the cage. At the end of the experiment, the rats were fasted for 12 h and then anesthetized by means of the intraperitoneal injection of sodium pentobarbital (60 mg/kg). Blood samples were collected for the biochemical assays. The left tibias were subjected to undecalcified section for bone histology. The right femurs and lumbar spines were used for the bone densitometry, then the right femurs were decalcified and used for the bone histomorphometry and immunohistochemistry assay. The left femurs were tested biomechanically prior to the analysis of the chemical component of calcium (Ca), phosphorus (P), and hydroxyproline (Hyp).

### Glucose tolerance test and insulin tolerance test

Insulin sensitivity was determined using the glucose tolerance test (GTT) and the insulin tolerance test (ITT) as described previously [20]. Briefly, following overnight fasting, the GTT assay was begun with the intraperitoneal injection of glucose at 2g/kg body weight. The ITT assay was conducted with the intraperitoneal injection of insulin at 1 U/kg body weight after fasting for 4–6 h. The blood glucose concentrations were measured from nicked tail veins at 0, 30, 60, 90 and 120 min using a OneTouch Ultra glucometer. The data were plotted as the blood glucose concentration over time.

### Serum markers assays

Blood was sterilely collected through the ventral aorta into specimen tubes and kept at 25°C for 1 h in a vertical position to allow for completely clotting. Next, the serum was separated by centrifuging at 1,000×g for 10 min and then stored at -80°C for the biochemical markers assays. The fasting blood glucose (FBG), triglyceride (TG), total cholesterol (TC), very-low-density lipoprotein cholesterol (VLDL-C), high-density lipoprotein cholesterol (HDL-C), and low-density lipoprotein cholesterol (LDL-C) levels were measured using an automatic biochemical analyzer (Olympus AU640). The serum estradiol (E2) was determined using radioimmunoassay (RIA), while the serum N-mid fragment of osteocalcin (N-mid osteocalcin, N-MID-OT) and tartrate-resistant acid phosphatase (TRAP) (both obtained from Yuanye Biological Technology, Shanghai, China) were assayed by ELISA according to the manufacturer’s instructions and the literatures [21, 22].

### Cortical bone observation and Masson-Goldner trichrome staining

The undecalcified (left) tibias were embedded in methyl methacrylate, and then stained for excessive collagen deposition using the Masson-Goldner trichrome technique [23]. The unstained 8-μm cross-sections of the middle tibias were photographed for the fluorescence and cortical bone analysis using a Nikon 80i microscope (Japan). The distance between the yellow line and the green line was measured in fluorescence labeling experiment.

### Bone densitometry determination

Right femurs and lumbar spines devoid of soft tissues were isolated for bone mineral density (BMD) and bone mineral content (BMC) determination according to the guidelines of the American Society for Bone and Mineral Research (ASBMR) using a dual-energy X-ray absorptiometry system (GE Healthcare Lunar iDXA, USA). The BMD measurements were performed at the midpoint of the bone and 2 cm proximal, and the BMD was calculated as the BMC/BW (bone width). Then, the right femurs were fixed with 4% paraformaldehyde for 48 h and decalcified with 13% EDTA for 2–3 months at room temperature, before being used for hematoxylin and eosin (H&E) staining and immunohistochemistry respectively.

### H&E staining and bone histomorphometry

The uterus of each rat was used to detect the influence of ovariectomy-induced estrogen deficiency by means of H&E staining. Following decalcification, the distal metaphyses of the right femurs were dehydrated in ethanol, defatted in xylene, and embedded in paraffin, before being sliced into longitudinal sections (5-μm thick). The H&E staining was performed on the longitudinal sections. The morphology of the rat trabeculae in the distal femoral metaphyses were observed, and the static parameters of the distal femoral cancellous bone histomorphometry were calculated using Image J software. The parameters measured were the percentage of the trabecular bone area (%Tb.Ar), trabecular bone thickness (Tb.Th), trabecular bone number (Tb.N), and trabecular bone separation (Tb.Sp).

### Immunohistochemistry

The specific biomarkers of the osteoblasts and osteoclasts were identified in the paraffin-embedded longitudinal femur sections by means of immunohistochemical analysis with mouse anti-osteocalcin and rabbit anti-MMP9 antibodies (both obtained from Beijing Biosynthesis Biotechnology, China), respectively. The method represented a modification of the manufacturers’ instructions [24]. Briefly, the endogenous peroxidase activity of the slides was blocked by 3% H_2_O_2_ while the nonspecific binding was blocked with 10% goat serum. The slides were then incubated with the primary antibody at 4°C overnight in a humidified chamber. The next day, the slides were incubated with the biotinylated secondary antibody for 10–15 min, before being incubated with streptavidin conjugated to horseradish-peroxidase for 15 min. A drop of freshly prepared DAB was applied on the slide for color development. The reaction was stopped when a uniform brown color became visible on the slide following rinsing in running water. Counterstaining was performed using hematoxylin for 5–10 seconds. A control experiment was performed by replacing primary antibody with PBS.

### Biomechanical test

Left femurs devoid of soft tissues were used to evaluate the effect of OVX or diet on the strength of the cortical bone using a three-point bending test performed with an INSTRON E3000 system (Instron, High Wycombe, UK). The femur was placed in a customized, three-point bending rig at a support width of 17 mm. A central bending load was then applied at a displacement rate of 1 mm/min, and the testing system was loaded on each bone in three-point bending rig until failure. The elastic load, maximum load, flexural strain, maximum strain, and flexural modulus were all calculated.

### Sample digestion and component analysis

After being subjected to the biomechanical test, the left femurs were dried at 80°C for 72 h and the ash weights were measured using an analytical balance. Then, the bones were digested using 6 mol/L hydrochloric acid in a 108°C oven for 24 h. The filter liquor was used for bone mineral and amino acid content analysis. The bone calcium (bone Ca) levels were determined using an atomic emission spectrometer (Optima 7000 DV, PerkinElmer CO., America), while the phosphorus (bone P) and hydroxyproline (bone Hyp) (both obtained from Jiancheng Bioengineering Institute, Nanjing, China) levels were measured using the molybdenum blue and p-dimethlaminobenzaldehyde colorimetric methods, respectively.

### Statistical analysis

All the values were expressed as the mean ± SD. The statistical analysis was performed using a two-way analysis of variance (ANOVA) with SPSS 16.0 software (Chicago, IL, USA). The statistics for the weight, GTT and ITT were obtained using a repeated measures two-way ANOVA. Levene’s test of the equality of error variances was first used to assess the results. If it showed equal variance, then Fisher’s protected least significant difference (PLSD) test was used, but if it showed heterogeneity of variance, then the Games-Howel test was used. P values of less than 0.05 were considered to be significant. In order to analyze the different contributory factors, the study groups were further divided into two groups according to whether or not the operation was performed, sham operation groups (SHAM, including the SSCD, SHFD and SHFDD groups) and ovariectomized groups (OVX, including the OVX, OVX-HFD, OVX-HFDD groups). Additionally, the study groups were further divided into three groups according to the diet: standard chow groups (SC, including the SSCD and OVX groups), HFD groups (including the SHFD and OVX-HFD groups), and HFDD groups (including the SHFDD and OVX-HFDD groups).

## Results

### Influence of a HFD and a HFDD on body weight

**Fig 1:**
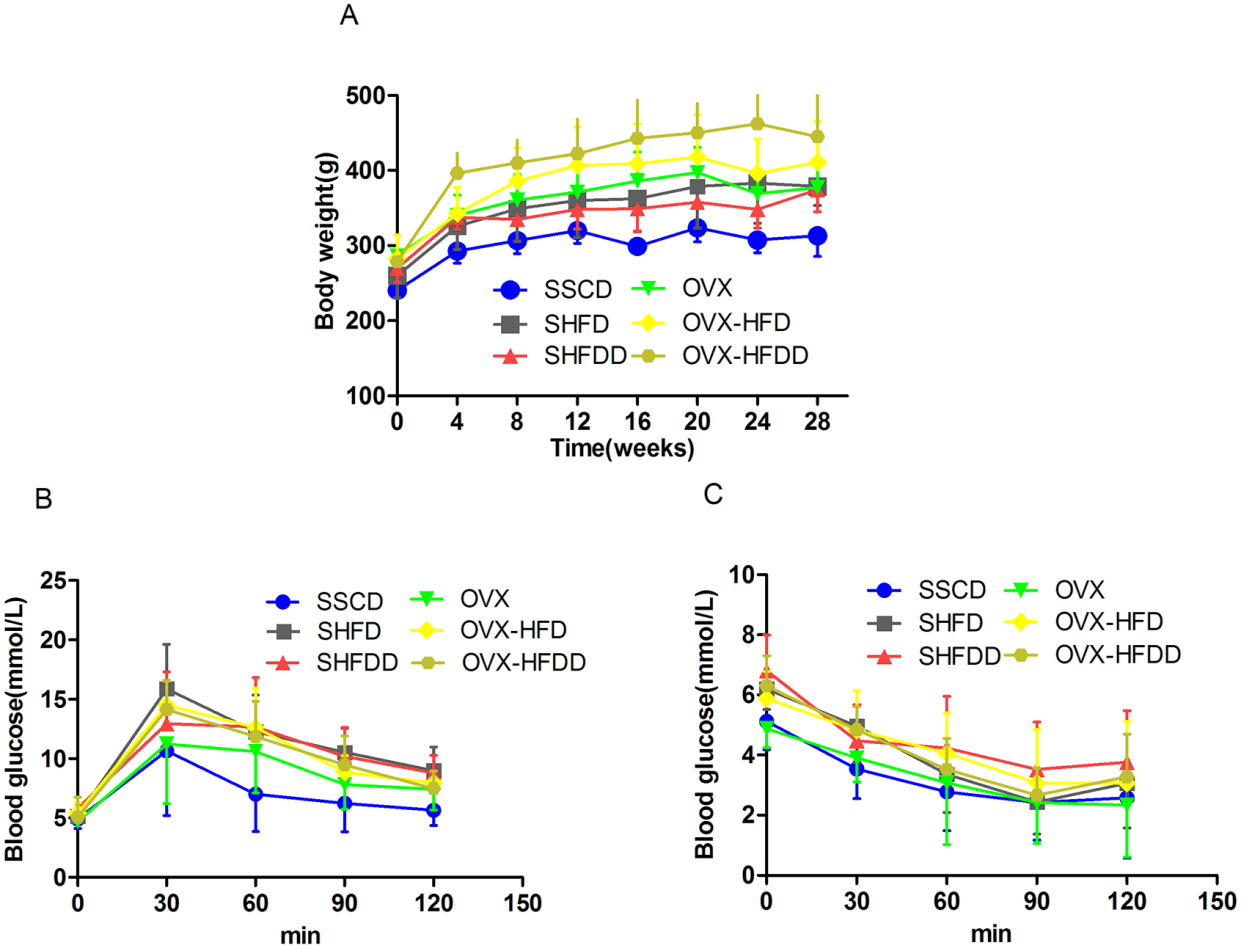
Changes of bodyweight, glucose and insulin sensitivity during the experimental period (*x*±*s*, n = 6). A. Changes of body weight; B. GTT (glucose tolerance test); C. ITT (insulin tolerance test). Blood samples were obtained from tail veins and measured using a glucometer; the data were plotted as blood glucose concentration over time. SSCD: sham-operated and standard chow diet, SHFD: sham-operated with HFD, SHFDD: sham-operated with HFDD; OVX: ovariectomized; OVX-HFD: OVX with HFD; OVX-HFDD: OVX with HFDD.

All the rats started the experiment with similar body masses, and the actual food intake showed no overt differences among the groups (*P*>0.05). The diversity of body weight between different time points had statistic significance (*P*<0.001), while the interaction between the time and the treatment factors also exhibited statistic significance. Further, a significant difference existed between the OVX and SHAM groups (*P*<0.001). The body weight of the HFD and HFDD groups were markedly higher than that of the SC groups (*P* = 0.003, *P*<0.001 respectively), although there was no difference between the HFD and HFDD groups (Fig 1A). These results indicated that a HFD and a HFDD promoted weight gain in OVX rats.

### Influence of a HFD and a HFDD on insulin resistance

As shown in Fig 1B, the blood glucose levels between the different time points during the GTT were significantly different (*P*<0.001). The interaction between the time and the treatment factors also were significantly different (*P* = 0.045). Further, the blood glucose levels in the HFD and HFDD groups were significantly higher than those in the SC groups (SSCD and OVX group) (*P* = 0.006, *P* = 0.014 respectively), although there was no difference between the HFD and HFDD groups (*P* = 0.303) (Table 1). Additionally, as shown in Fig 1C, the blood glucose levels between the different time points during the ITT were significantly different (*P*<0.001), although there was no difference between the OVX and SHAM groups or among the different diet groups. Combined with increased blood glucose levels, increased body weight and physical inactivity which were observed in the experiment, insulin resistance was successfully established in the model rats by both HFD and HFDD feeding.

**Table 1:** Effects of high-fat diets on serum indices of 13-month-old rats ($\bar{x}\pm s$).

Parameter	SSCD	SHFD	SHFDD	OVX	OVX-HFD	OVX-HFDD	*F* value	*P* value
	n = 6	n = 6	n = 6	n = 6	n = 6	n = 6		
N-MID-OT(ng/ml)	3.55±1.11	3.25±0.49	3.56±0.40	3.40±0.65	4.43±0.73	4.42±0.84	A:1.423	A:0.258
							B:6.325	B:0.018
							A*B:2.512	A*B:0.099
TRAP(U/L)	19.04±2.36	19.28±1.97	21.95±1.91	24.50±3.27	27.37±3.80	27.78±4.48	A:2.588	A:0.093
							B:34.884	B:<0.001
							A*B:0.586	A*B:0.563
TC(mmol/L)	1.66±0.32	1.99±0.35	2.42±0.47	2.37±0.50	2.06±0.23	2.28±0.21	A:2.993	A:0.066
							B:2.901	B:0.100
							A*B:4.205	A*B:0.025
TG(mmol/L)	0.44±0.15	0.52±0.38	0.74±0.39	0.46±0.09	0.60±0.30	0.75±0.41	A:2.537	A:0.097
							B:0.100	B:0.754
							A*B:0.041	A*B:0.960
LDL-C(mmol/L)	0.83±0.47	0.83±0.62	1.46±0.46	1.43±0.50	0.77±0.47	1.24±0.65	A:3.030	A:0.064
							B:0.337	B:0.566
							A*B:1.895	A*B:0.169
HDL-C(mmol/L)	0.76±0.21	0.81±0.43	0.69±0.25	0.77±0.15	1.21±0.52	0.76±0.67	A:1.682	A:0.204
							B:1.303	B:0.263
							A*B:0.785	A*B:0.466
VLDL-C(mmol/L)	0.24±0.07	0.32±0.18	0.27±0.14	0.17±0.03	0.33±0.11	0.28±0.15	A:3.139	A:0.059
							B:0.164	B:0.688
							A*B:0.317	A*B:0.731
E_2_(pmol/L)	128.67±28.35	157.00±25.31	151.80±27.37	89.17±27.64	103.33±38.75	111.60±36.31	A:1.959	A:0.160
							B:17.412	B:<0.001
							A*B:0.195	A*B:0.824
FBG(mmol/L)	8.98±2.67	14.44±2.39	13.17±3.15	10.45±1.96	13.02±2.12	11.97±3.21	A:7.629	A:0.002
							B:0.186	B:0.669
							A*B:1.141	A*B:0.334

A:effects of diet, B:effects of operation, A*B:interaction between diet and operation

### Influence of a HFD and a HFDD on plasma lipid, estrogen, and the bone metabolism indices and uterus morphology

**Fig 2:**
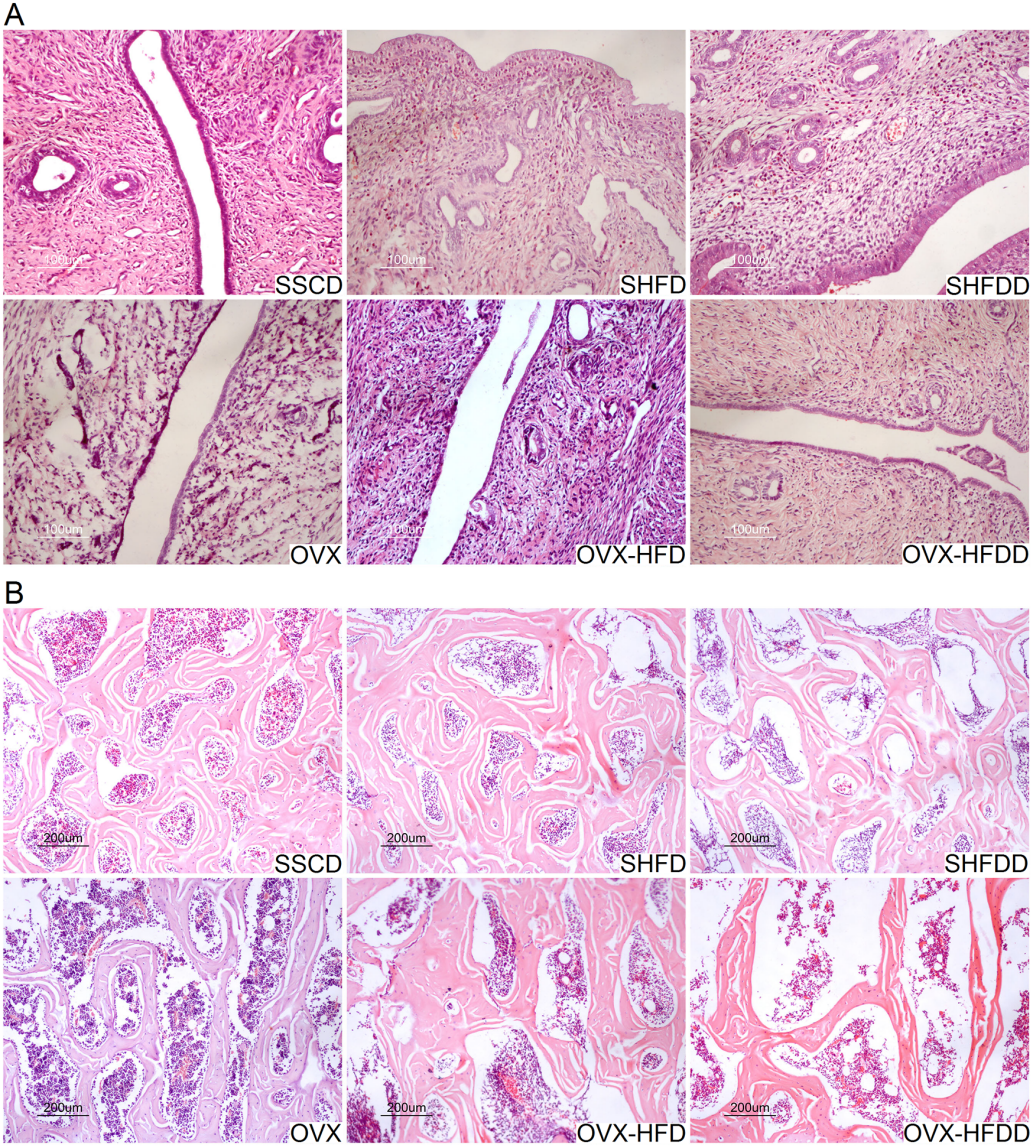
Effects of OVX, HFD and HFDD on the uterine and femoral pathology. A. H&E staining of the uterine (×200); B. H&E staining of the right femur (×200).

As shown in Table 1, when compared with SHAM groups, the serum N-MID-OT and TRAP levels in the OVX groups were obviously higher except for N-MID-OT levels in OVX group (*P* = 0.018 and *P*<0.001, respectively), although there was no difference among the different diet groups. There were also no differences in the serum TG, LDL-C, HDL-C and VLDL-C levels between the SHAM and OVX groups, or between the different diet groups. There were interactions in the TC levels between the diet and OVX groups. The serum E2 levels in the OVX groups were lower than those in the SHAM groups (*P*<0.001), while the uterine gland in the OVX, OVX-HFD, and OVX-HFDD group shrank considerably, and the volume of the uterus diminished following the OVX operation (Fig 2A), which indicates the success of the ovariectomy. There were no differences in the serum E2 levels between the different diet groups. These findings suggested that the OVX groups had a higher bone turnover rate when accompanied with HFD or HFDD feeding.

### Influence of a HFD and a HFDD on histological changes and histomorphometry

The H&E staining of the decalcified femurs is shown in Fig 2B. The structure of the bone trabeculae exhibited disorder, loosening, and breakage, and it was diminished in the OVX, OVX-HFD, and OVX-HFDD groups. Further, the marrow cavities became enlarged and the bone trabeculae in the SHFDD groups became more slender. The bone trabeculae in the OVX-HFDD group became the thinnest, while the marrow cavities in the three OVX groups became the largest (Fig 2B).

Additionally, the calculation and quantification of the H&E staining is shown in Table 2. When compared with the SHAM group, %Tb.Ar and Tb.Th in the OVX groups were obviously lower (*P*<0.001), while the %Tb.Ar and Tb.Th in the HFD groups were significantly higher than those in the SC and HFDD groups (*P*<0.001). The %Tb.Ar in the HFDD groups was significantly lower than that in the SC groups (*P* = 0.015), although there was no significant difference in terms of the Tb.Th between the HFDD and SC groups (*P* = 0.760). When compared with the SHAM groups, the Tb.N in the OVX groups exhibited no significant difference (*P* = 0.717), although the Tb.Sp in the OVX groups was higher (*P*<0.001). The Tb.N in the HFD groups was lower than that in the SC and HFDD groups (*P*<0.001), although no difference was observed between the SC and HFDD groups (*P* = 0.334). Interaction existed between the diet and the operation treatment. The minimum value of the %Tb.Ar (*P*<0.05) and the maximum increase in the Tb.Sp (*P*< 0.05) was observed in the OVX-HFDD group, which meant that the OVX combined with the HFDD treatment attenuated bone structure.

**Table 2:** Effects of high-fat diets on distal femoral metaphysis cancellous bone static parameters.

Parameter	SSCD	SHFD	SHFDD	OVX	OVX-HFD	OVX-HFDD	*F* value	*P* value
	*n* = 6	*n* = 6	*n* = 6	*n* = 6	*n* = 6	*n* = 6		
%Tb.Ar	48.85±6.16	63.22±5.43	40.47±6.81	37.59±4.35	41.98±3.55	34.79±5.96	A:22.318	A:<0.001
							B:47.715	B:<0.001
							A*B:6.068	A*B:0.006
Tb.Th(μm)	98.76±17.61	216.06±45.02	94.75±16.97	81.21±14.89	102.31±11.44	80.02±28.81	A:30.237	A:<0.001
							B:31.912	B:<0.001
							A*B:14.493	A*B:<0.001
Tb.N(mm^-1^)	4.91±0.51	3.05±0.43	5.19±0.93	4.73±0.53	4.14±0.37	4.08±0.55	A:16.233	A:<0.001
							B:0.134	B:0.717
							A*B:10.930	A*B:<0.001
Tb.Sp(μm)	108.06±10.50	123.38±21.51	120.06±12.95	135.23±17.48	141.71±14.99	159.09±22.52	A:3.126	A:0.059
							B:22.994	B:<0.001
							A*B:1.021	A*B:0.373

A:effects of diet, B:effects of operation, A*B:interaction between diet and operation

### Influence of a HFD and a HFDD on cortical bone changes in tibia tissue

**Fig 3:**
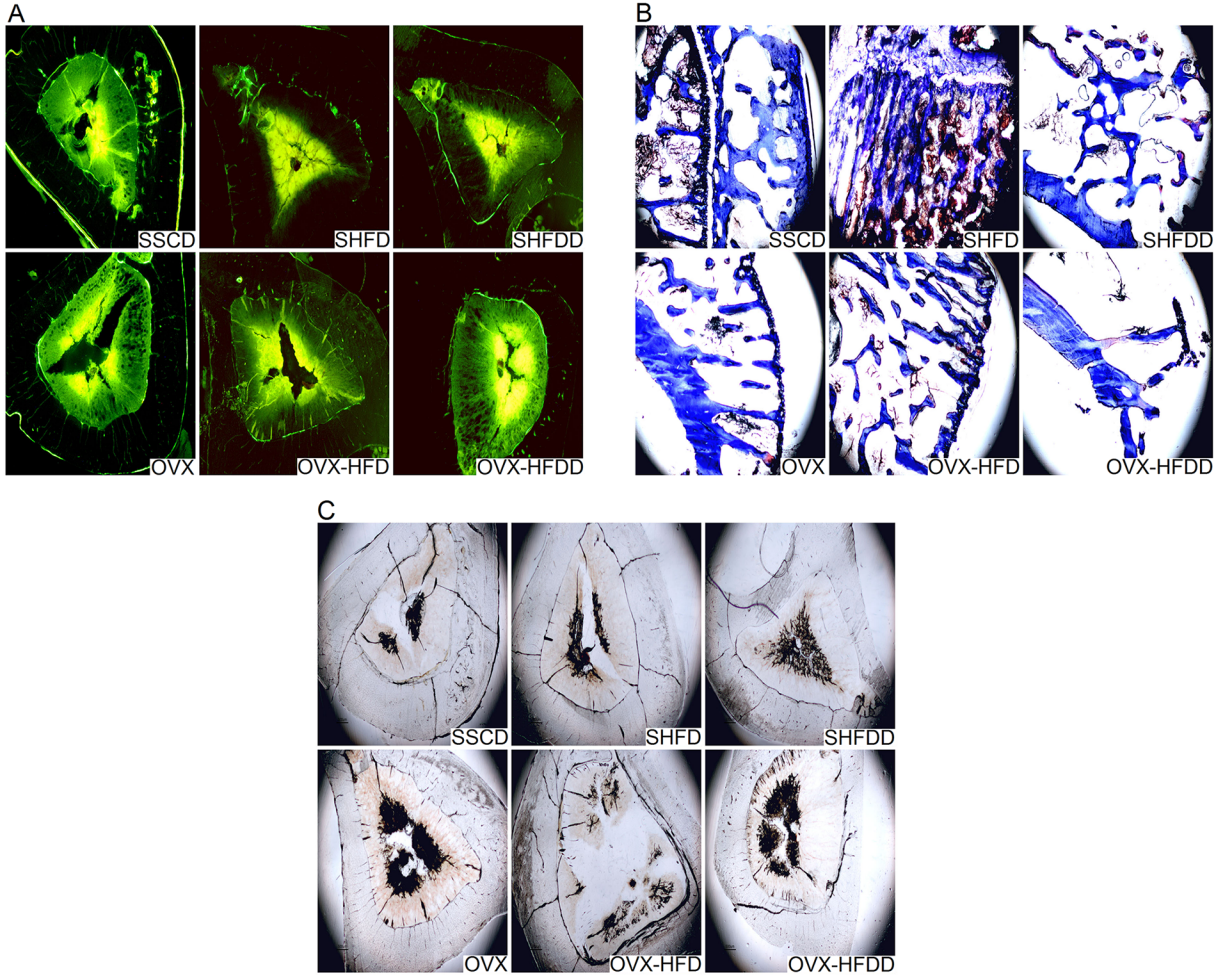
Effects of OVX, HFD and HFDD on histological change in tibia tissue. A. Fluorescence staining in tibia tissue. (×40); B. Masson-Goldner trichrome staining in tibia tissue (×40); C. undyed cortical bone change in tibia tissue (×40).

The marrow cavities became larger and the area of the cortical bone was decreased in the OVX group, with the most obvious changes being seen in the OVX-HFDD group by means of fluorescence and undyed analysis (Fig 3A and 3C). As the rats had reached a sufficient age when the experiment ended, the new bone formation had slowed down and became indistinct, and we didn’t observe any differences in the distance between the yellow line and the green line in the fluorescence labeling experiment (results not shown). The results of the Masson-Goldner trichrome staining showed that the bone trabeculae became sparse, fractured, thinned, and depleted in numbers in the OVX and OVX-HFDD groups. HFD feeding rendered the bone trabeculae bulky, thereby having a protective effect on the bone, while a HFDD had a deleterious effect (Fig 3B).

### Influence of a HFD and a HFDD on the BMD and BMC

Table 3 showed the influence of diet on the BMD and BMC of the rats’ femurs and lumbar spines. As compared with the SHAM groups, the BMD of the right femur in the OVX groups was significantly lower (*P*<0.001). The BMD of the right femur in the SC and HFD groups were significantly higher when compared with that of the HFDD groups (*P* = 0.018 and *P*<0.001, respectively), although there was no difference between the HFD and SC groups (*P* = 0.101). As compared with the SHAM groups, the BMC of the right femur in the OVX groups was significantly lower (*P* = 0.004). The BMC of the right femur in the HFD groups was obviously higher than that of the SC and HFDD groups (*P* = 0.009 and *P* = 0.002, respectively), and there was no difference between the SC and HFDD groups (*P* = 0.403). There was interaction between the operation treatment and diet. When compared with the SHAM groups, the BMD and BMC of the lumbar spines in the OVX groups were significantly lower (P<0.001 and *P* = 0.006, respectively), while the BMD and BMC of the lumbar spines in the HFD groups were significantly higher than those in the SC and HFDD groups (both *P*<0.001; *P* = 0.011 and *P* = 0.022, respectively). Further, there was no difference between the SC and HFDD groups (*P* = 0.169 and *P* = 0.819, respectively). All these results implied that the OVX operation decreased the BMD and BMC in the right femur and lumbar spines, while a HFD inverted the decreased BMD and BMC caused by the OVX, and a HFDD reduced the BMD and BMC in the femur and lumbar spines.

**Table 3:** Effects of high-fat diets on the BMD and BMC of right femurs and lumbar vertebras in 13-month-old rats ($\bar{x}\pm s$).

Parameter	SSCD	SHFD	SHFDD	OVX	OVX-HFD	OVX-HFDD	*F* value	*P* value
	n = 6	n = 6	n = 6	n = 6	n = 6	n = 6		
femur BMD(g/cm^2^)	0.261±0.066	0.300±0.023	0.232±0.026	0.221±0.016	0.223±0.018	0.181±0.023	A:8.576	A:0.001
							B:26.398	B:<0.001
							A*B:0.990	A*B:0.383
femur BMC(g)	0.669±0.302	1.429±0.363	0.519±0.127	0.518±0.125	0.814±0.404	0.471±0.082	A:19.895	A:<0.001
							B:9.823	B:0.004
							A*B:3.991	A*B:0.029
Lumbar vertebra BMD(g/cm^2^)	0.198±0.019	0.312±0.037	0.195±0.051	0.200±0.017	0.204±0.029	0.164±0.045	A:16.713	A:<0.001
							B:15.685	B:<0.001
							A*B:8.074	A*B:0.002
Lumbar vertebra BMC(g)	0.554±0.147	0.849±0.095	0.631±0.251	0.545±0.099	0.567±0.104	0.495±0.139	A:4.322	A:0.022
							B:8.571	B:0.006
							A*B:2.658	A*B:0.086

A:effects of diet, B:effects of operation, A*B:interaction between diet and operation

### Influence of a HFD and a HFDD on bone strength

Trabecular and cortical bone abnormalities are known to be related to the strength of tissues; hence, the bone strength of the femurs was assessed (Table 4). When compared with the SHAM groups, the elastic load and maximum load in the OVX groups were obviously lower (P = 0.001 and P = 0.009, respectively). The elastic load and maximum load in the HFD groups were significantly higher than those in the SC and HFDD groups (*P* = 0.005 and *P*<0.001; *P* = 0.001 and *P*<0.001), while there were no differences between the SC and HFDD groups (*P* = 0.285 and *P* = 0.231, respectively). There existed interaction between the diet and operation groups. There were no differences in the flexural strain, maximum strain, and flexural modulus between the SHAM and OVX groups, and there were also no differences between the different diet groups. These findings indicated that a HFD increased bone strength, while a HFDD decreased bone strength; hence, the bones in the HFDD groups were fragile and prone to fracture.

**Table 4:** Effects of high-fat diets on bone strength of left femurs in 13-month-old rats ($\bar{x}\pm s$).

Parameter	SSCD	SHFD	SHFDD	OVX	OVX-HFD	OVX-HFDD	*F* value	*P* value
	n = 6	n = 6	n = 6	n = 6	n = 6	n = 6		
Elastic load (N)	115.8±16.8	161.6±16.0	125.1±31.9	115.3±14.0	125.6±27.6	81.8±16.1	A:10.257	A:<0.001
							B:12.500	B:0.001
							A*B:3.018	A*B:0.065
Maximum load (N)	150.1±11.3	208.6±12.7	159.8±32.3	161.6±17.5	169.3±21.6	125.5±25.4	A:14.405	A:<0.001
							B:7.919	B:0.009
							A*B:4.799	A*B:0.016
Flex strain(mm)	0.27±0.09	0.36±0.13	0.47±0.16	0.29±0.12	0.39±0.13	0.36±0.15	A:2.821	A:0.077
							B:0.207	B:0.652
							A*B:0.953	A*B:0.398
Maximum strain(mm)	0.48±0.13	0.64±0.14	0.69±0.18	0.60±0.21	0.70±0.20	0.62±0.20	A:1.813	A:0.182
							B:0.412	B:0.526
							A*B:0.804	A*B:0.458
flex modulus(Mpa)	7822±2021	6621±1579	6522±322	5299±2388	5775±1267	6734±1368	A:0.242	A:0.787
							B:3.650	B:0.066
							A*B:2.026	A*B:0.151

A:effects of diet, B:effects of operation, A*B:interaction between diet and operation

### Influence of a HFD and a HFDD on bone mineral content and organic matrix

**Fig 4:**
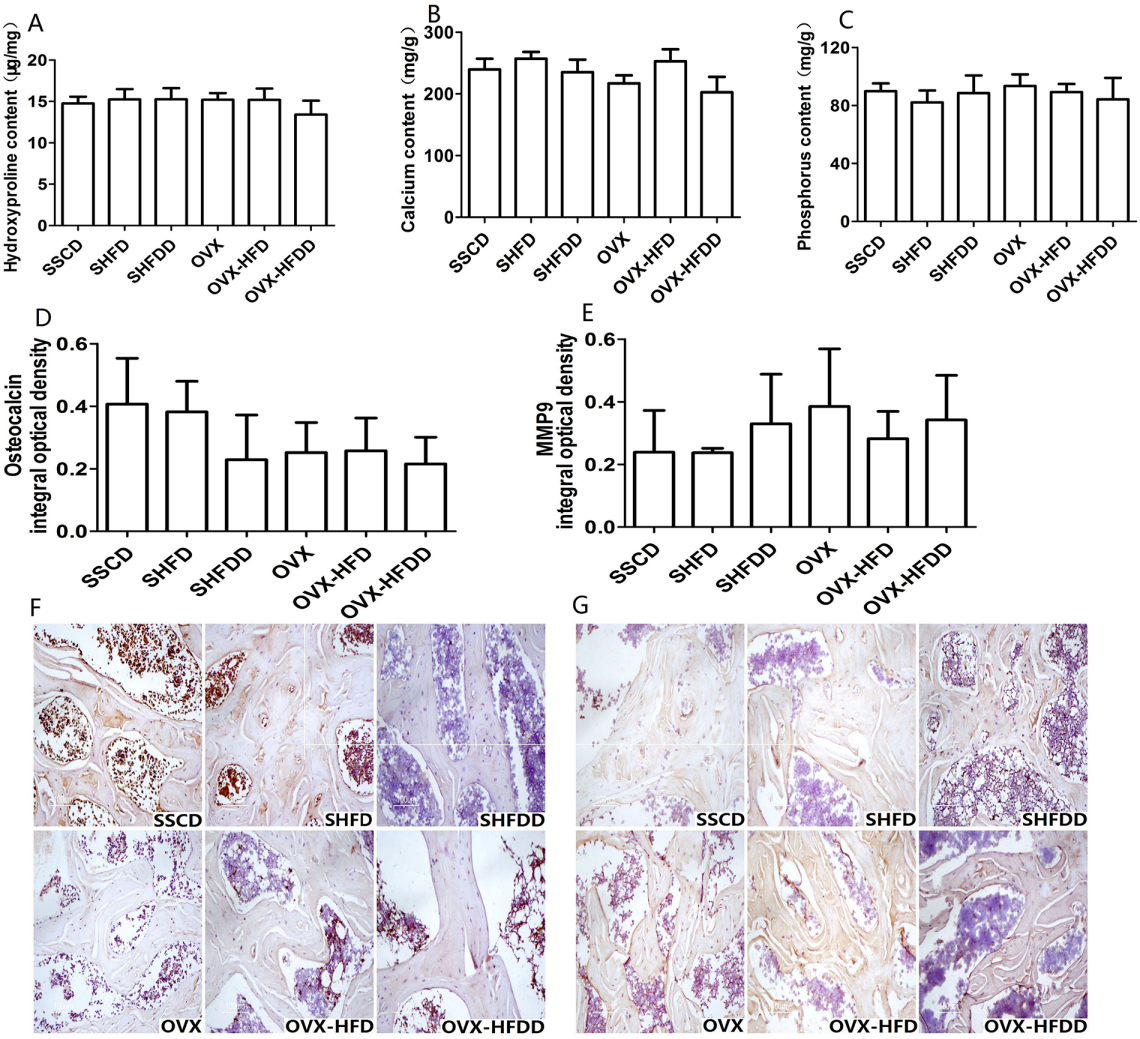
Effects of OVX, HFD and HFDD on contents of hydroxyproline, calcium, and phosphorus, expression of osteocalcin and MMP9 protein in femur (*x*±*s*, n = 6). A. levels of hydroxyproline; B. levels of calcium; C. levels of phosphorus; D. quantification of protein expression of osteocalcin; E. quantification of protein expression of MMP9; F. expression of osteocalcin (IHC staining, ×200); G. expression of MMP9 (IHC staining, ×200).

The bone organic matrix and mineral contents in rats are presented in Fig 4A, 4B and 4C. There were no differences in the bone Hyp and P levels in the femurs between the SHAM and OVX groups, while there were also no differences between the different diet groups. The bone Ca levels of the femurs in the OVX groups were markedly lower than those in the SHAM groups (*P* = 0.003), while the bone Ca levels in the HFD groups were significantly higher than those in the SC and HFDD groups (*P* = 0.002 and *P*<0.001, respectively), although there was no difference between the SC and HFDD groups (*P* = 0.208). These results suggested that HFD feeding increased the bone Ca levels.

### Influence of a HFD and a HFDD on protein expression in bone tissues

The cellular and macromolecular composition of the bone matrix was assessed using osteocalcin and MMP9 immunohistochemistry, respectively. The osteocalcin protein expression in the OVX groups was obviously lower than that in the SHAM groups (*P* = 0.01) (Fig 4D and 4F), while the MMP9 protein expression exhibited no differences between the OVX and SHAM groups (Fig 4E and 4G). There were no differences between the different diet groups. These results confirmed that the numbers of osteoblasts were markedly decreased in the femurs of the OVX groups, including the OVX, OVX-HFD, and OVX-HFDD groups.

## Discussion

The increasing prevalence of both obesity and osteoporosis in the general population along with the controversial findings concerning the relationship between the two conditions suggest the need for further investigation. Obesity is a major public health concern as well as a risk factor for many diseases, but determining whether it is detrimental or beneficial to bone health is difficult. It is important to note that some inconsistencies regarding how a high-fat diet affects bone can be attributed to differences in study design (i.e., feeding ingredients, age at initiation and duration of treatment, gender, strain/substrain, etc.).

In our experiment, the ovariectomy approach was applied in 6-month old female SD rats. Then, the ovariectomized rats were fed a standard HFD (D12451) or HFDD for seven months in order to study the influence of the diets on bone parameters, including the femurs, tibias, and lumbar spines. Thus, we are able to show for the first time that the ovariectomized rats fed with a D12451 increased their body mass as well as the microstructural and mechanical properties of their long bones. However, when vitamin D, phosphorus, and calcium were deleted from the D12451, the diet-mediated osteoporosis-preventive effects vanished. These results showed that our hypothesis was correct.

Typically, 3-month-old female rats were selected as experimental subjects. We instead selected 6-month-old female SD rats as the subjects of ovariectomy, since the rats were older and, after OVX and seven months of HFD or HFDD feeding, they were approaching old age judged by atrophied uterus although they were only 13-month-old [25], which meant that they could more accurately physiologically mimic PMOP. A previous study has reported the use of similarly aged rats in an ovariectomy experiment [26].

The standard D12451 is composed of casein, corn starch, maltodextrin, sucrose, soybean oil, lard, and a mineral and vitamin mix. Since 45% of energy comes from fat in the diet (soybean oil and lard), it is considered to be a typical high-fat diet [19]. The difference between the HFD and HFDD conditions was simply the presence or lack of vitamin D3, phosphorus, and calcium, so both conditions should promote weight gain in the SHAM or OVX groups. Indeed, our results confirmed the above speculation. Ovariectomy results in body weight gain, while estradiol administration results in a reversal of that weight gain [27], so the OVX-HFD or OVX-HFDD condition combined the effects of OVX and HFD or HFDD and the rats exhibited the highest body mass. Our study regarding body mass suggested that estrogen deprivation leads to obesity in rodents, while the HFD and HFDD resulted in further weight gain (Fig 1A).

Vitamin D3, phosphorus, and calcium deficiencies are the main factors behind osteoporotic fractures among elderly women. Parameswari et al. reported that ovariectomized rats fed with a diet deficient in calcium, phosphorus, and vitamin D showed severe bone loss and bone resorption [28], which was similar to the findings of our study, although the diet in their study was a normal diet rather than a HFD. Therefore, therapeutic strategies designed to improve lifestyle measures for the prevention and treatment of osteoporosis should include calcium, phosphorus and vitamin D supplementation.

The serum E2 levels of the OVX rats decreased significantly, which was confirmed by the atrophied uterine gland in the OVX rats (Fig 2A), while the HFD or HFDD increased the E2 levels to some degree in the OVX rats, which maybe came from expanded adipose tissue or other tissues [29], although they were still lower than in the SSCD, SHFD, and SHFDD groups.

Serum osteocalcin is easily degraded, so N-MID-OT is often detected as a bone turnover marker because of its good stability and high sensitivity, with the levels reflecting the activity and status of the newly formed osteoblasts [30]. Our results indicated that the OVX-HFD and OVX-HFDD groups have increased serum N-MID-OT and TRAP levels accompanied by insulin resistance, although the consequences of insulin resistance for bone remain largely unknown. This was inconsistent with the results of Tonks et al. [31], who reported that increased visceral adiposity and higher fasting insulin in insulin-resistant states are associated with lower fasting osteocalcin and a failure to further suppress it with more insulin. Recently, a study found that in nondiabetic, postmenopausal women, insulin resistance was associated with smaller bone size, greater volumetric bone mineral density, and generally favorable bone microarchitecture at both weight-bearing and nonweight-bearing skeletal sites [32]. Our findings about the OVX-HFD rats were basically consistent with those results.

There were some inconsistency in terms of the levels of serum N-MID-OT and osteocalcin expression in the femur detected by immunohistochemistry. The serum N-MID-OT levels in the OVX-HFD and OVX-HFDD groups were increased (Table 1), while the expression of osteocalcin in the femoral trabecular bone of the SHFDD and three OVX groups were decreased (Fig 4D and 4F). The serum N-MID-OT comes from all bones and it reflects the total expression levels, but we only detected the expression of osteocalcin in the femoral bone, so the bones in other sites perhaps have contributed to the increased serum N-MID-OT levels.

The SHFD, SHFDD, OVX, OVX-HFD and OVX-HFDD rats exhibited elevated FBG levels (Table 1), indicating that the model of PMOP combined with glucose metabolic disturbances in rats was successfully established, which is consistent with the results of Sugimoto [33]. Ovariectomy (estrogen depletion) in rats leads to glucose intolerance and insulin resistance, and these complications were further augmented when the ovariectomy was followed by a HFD, especially HFDD feeding. However, it remains unclear why the serum lipid indices in the HFD and HFDD groups were not influenced by the high-fat diet, although this finding could be related to the poor lipid-metabolic ability of SD rats.

Again, the OVX-HFDD group showed a significant decrease in the %Tb.Ar, Tb.N and Tb.Th as well as increased Tb.Sp in the femur (Table 2), which meant that the bone became scarce following the joint treatment of OVX and HFDD feeding. The results of the H&E, undecalcified, fluorescence, and Masson-Goldner trichrome staining exhibited decreased trabecular thickness, enlarged marrow cavities, and a disordered trabecular structure, all of which reflected the morphological changes in the tibia and supported the data obtained from the bone histomorphometry. The structural damage to the trabecular bone in the OVX-HFDD group was the most serious of that seen in the three OVX groups.

The BMD and BMC are important indicators in the clinical diagnosis of osteoporosis. When 12-month-old female C57BI/6J mice were fed a HFD containing 10% corn oil for six months, the BMD of the femur and tibia decreased [34]. Ok HM et al. also found that the femoral BMD of 8-week-old ovariectomized SD rats decreased significantly following feeding with a HFD (corn oil plus lard as dietary fat source) for eight weeks [35].Our result was different from those reports, which showed that the levels of BMD and BMC in the femur and lumbar vertebra as well as the femoral Ca content and bone mass in the OVX-HFD (soybean oil plus lard as dietary fat source) group were significantly increased as compared with the OVX rats. The reason was possibly related to the soybean oil, which was rich in omega-3 fatty acids and known to have the protective effect on osteoporosis [36]. The HFDD reduced the BMD and BMC levels in the femur and lumbar vertebra, and it also reduced the femoral Ca in both the SHFDD and OVX-HFDD groups, which suggested that a HFDD aggravates bone mass loss in normal and OVX rats (Table 3, Fig 3B).

The three-point bending test is a commonly used method for measuring bone biomechanical properties in animal experiments. In this study, the bone strength reduced significantly in the OVX rats. The HFD increased the bone strength of the normal and OVX rats, while the HFDD decreased the bone strength of the normal and OVX rats (Table 4). The changes in bone mass (i.e., BMD and BMC) and bone strength seen in the obese rats supported our expectation that long-term D12451 supplementation improves bone quality, while a long-term HFDD lowers the bone quality and weakens the bone strength of OVX rats.

Using PMOP rats, it has previously been shown that this may be due to a nutritionally balanced HFD causing an increase in bone mass, which enhances osteoblast activity and thus bone growth, conversely a nutritionally deficient HFDD decreases bone mass and osteoblast activity [37]. A novel finding suggested by our experiment is that the influences of a HFD on bone in POMP rats is related to the ingredients of the HFD—especially calcium, phosphorus, and VitD3—which indicates a theoretical basis for dietary intervention in osteoporosis.

Melhus et al reported that a HFD coupled with nutrient deficiency might accelerate the bone turnover rate by activating the parathyroid in OVX rats, thereby further aggravating bone mass loss [38]. The levels of parathyroid should therefore be detected in an extension of our experiment. Additionally, the molecular mechanism of the interaction between obesity and PMOP should be studied in vivo and in vitro.

In conclusions, the present study described how a standard HFD increased the bone strength of OVX rats and as a beneficial factor for bone, while a HFDD aggravated bone loss and decreased bone strength in OVX rats, which may be related to the deficiency in calcium, phosphorus, and vitamin D3 found in that diet.

## Supporting information

S1 Fig Changes of bodyweight, glucose and insulin sensitivity during the experimental period. A.Body weight in six study groups; B. GTT in six study groups;C. ITT in six study groups.
(RAR)

S2 Fig File for Fig 4. **Effects of OVX, HFD and HFDD on contents of hydroxyproline, calcium, and phosphorus, expression of osteocalcin and MMP9 protein in femur**. A.Bone hydroxyproline levels in left femur of six study groups; B. Bone calcium levels in left femur of six study groups; C. Bone phosphorus levels in left femur of six study groups; D. Immunohistochemical analysis for osteocalcin in right femur of six study groups; E. Immunohistochemical analysis for MMP9 in right femur of six study groups.
(RAR)

S1 Table Effects of high-fat diets on serum indices of 13-month-old rats. 1. Serum biochemical markers and estrodiol levels in six study groups; 2. Serum N-MID-OT and TRAP levels in six study groups.
(RAR)

S2 Table Histomorphometry of right femur in six study groups. (XLS)

S3 Table Effects of high-fat diets on the BMD and BMC of right femurs and lumbar vertebras in 13-month-old rats. 1.Femur BMD and BMC in six study groups; 2. Lumbar vertebra BMD and BMC in six study groups.
(RAR)

S4 Table Bone strength in left femur of six study groups. (XLS)
